# Influence of brief carbon dioxide inhalation on acute exercise performance and recovery: A pilot study

**DOI:** 10.14814/phy2.70893

**Published:** 2026-06-19

**Authors:** Dongwook Yeo, Jesse C. Schwartz, Bruce D. Johnson, Chul‐Ho Kim

**Affiliations:** ^1^ Department of Cardiovascular Diseases, Mayo Clinic Human Integrative and Environmental Physiology Laboratory Rochester Minnesota USA

**Keywords:** carbon dioxide, cardiovascular recovery, chemoreflex, exercise tolerance, high intensity interval exercise, hypercapnia

## Abstract

Brief inhalation of carbon dioxide (CO_2_) has been proposed to counteract transient hypocapnia by enhancing oxygen unloading, vasodilation, and sympathetic modulation. Despite these potential ergogenic mechanisms, CO_2_ has rarely been investigated in the exercise context. The present study investigated whether brief CO_2_ inhalation (iCO_2_) influences performance and recovery during high‐intensity interval exercise. Nine healthy adults completed two randomized, single‐blinded crossover trials of repeated cycling intervals at 85% peak work rate until volitional exhaustion or cadence dropped below 60 rpm, with a minimum 2‐min interval duration required and 3‐min active recovery between bouts. Participants inhaled 5% CO_2_ or room air (15 breaths) before and after each exercise bout. Gas exchange, cardiovascular responses, and perceived exertion were assessed. Total exercise time was similar between trials; however, participants completed more intervals with iCO_2_. During the first interval, iCO_2_ increased exercise duration and VO_2_. At matched exercise time, perceived exertion and dyspnea were lower with iCO_2_. During recovery, systolic blood pressure was lower and heart rate was higher following iCO_2_. Brief iCO_2_ improved first‐interval performance, altered cardiovascular recovery, and reduced perceptual strain but did not extend overall exercise duration. These findings suggest iCO_2_ may transiently modulate tolerance and autonomic‐vascular responses during high‐intensity interval exercise.

## 1 INTRODUCTION

Carbon dioxide (CO_2_) is among the most prevalent gases in the atmosphere and plays a significant role in maintaining physiological homeostasis. Furthermore, CO_2_ is a byproduct of cellular metabolism and can trigger a cascade of physiological responses, including acid–base balance, vascular tone, neural activity, and cellular signaling (Hoiland et al., 2019; Talabko et al., 2023). Forms of brief CO_2_ inhalation (iCO_2_) have worked their way into various performance‐related activities, including hypotheses around recovery or windows when individuals may transiently be hypocapnic. Accordingly, CO_2_ is a significant physiological factor, and its importance becomes particularly pronounced during exercise, when metabolic demands and respiratory activity are elevated.

Elevated levels of CO_2_ contribute to increased blood acidity, thereby inducing a rightward shift of the oxyhemoglobin dissociation curve (Bohr effect). This shift reduces hemoglobin's affinity for oxygen (O_2_), facilitating enhanced O_2_ unloading into the tissues (Benner et al., 2018; Woyke et al., 2021). Additionally, elevated CO_2_ concentrations promote vasodilation through the endothelial production of nitric oxide (NO), thereby improving regional blood flow (Carr et al., 1993; Hewitt et al., 1973). Furthermore, CO_2_ activates central and peripheral chemoreceptors, potentially modulating sympathetic nervous system activity, and this may help maintain cardiovascular outflow during physical exertion (Jackson et al., 1974). CO_2_ may also have potent anti‐inflammatory properties and act as an inhibitor of reactive oxygen species (Bolevich et al., 2016; Galganska et al., 2023). Taken together, these mechanisms suggest that CO_2_ may exert ergogenic effects on exercise performance and/or recovery; however, this hypothesis remains to be clearly tested.

For the present study, an intense interval exercise protocol was utilized. This type of exercise protocol can provide a robust framework for evaluating overall aerobic performance, as alternating periods of high‐intensity work and recovery allow comprehensive assessment of cardiovascular and respiratory capacity, fatigue, and recovery within a single session. Accordingly, the present study investigated if iCO_2_ improved exercise performance during interval exercise. We hypothesized that iCO_2_ would improve exercise performance and recovery compared with control conditions.

## 2 METHODS

### 2.1 Participants

Eleven healthy adults participated in the present study. Participants were recruited from the Mayo Clinic community and the Rochester, MN area through classified advertisements, email announcements, and word of mouth. All participants were free of known cardiovascular or pulmonary disease and engaged in regular moderate‐ to high‐intensity exercise (3–5 days per week). Inclusion criteria required that participants had a maximal oxygen consumption (VO_2_max) greater than 120% of the age‐predicted value. Individuals with musculoskeletal disorders or injuries were excluded. Participant characteristics are summarized in Table 1.

**TABLE 1:** Participant characteristics.

Variables	Values
Sex (male/female)	4/5
Age (years)	32.4 ± 8.7
Height (cm)	171.8 ± 10.2
Weight (kg)	68.2 ± 11.9
BMI (kg/m^2^)	22.9 ± 2.2
VO_2_max (ml/kg/min)	48.7 ± 3.7
% age‐predicted VO_2_max (%)	126.0 ± 7.7
Maximal Workload (W)	239.4 ± 52.0
85% maximal workload (W)	203.7 ± 44.3

*Note*: Data are presented as mean ± SD. Maximal oxygen consumption (VO_2_max) and peak workload were obtained from the maximal exercise test, and 85% maximal workload represents the intensity used during interval exercise.

Abbreviation: BMI, body mass index.

This study protocol was approved by the Mayo Clinic Institutional Review Board (Protocol No. 23‐012477) and written informed consent was obtained from all participants prior to participation.

### 2.2 Study design

The present study employed a single‐blinded, randomized, and crossover study design. Participants visited the Human Integrative and Environmental Physiology laboratory at Mayo Clinic on three separate occasions: one screening visit and two experimental visits consisting of a treatment trial (iCO_2_: 5% CO_2_) and control trial (CON: room air). The order of trials was randomized, and each visit was separated by a minimum 72‐h washout period.

Participants were asked to refrain from caffeine and vigorous exercise at least 24 h prior to each visit. During the screening visit, informed consent was obtained, and individuals of childbearing potential underwent a urine pregnancy test.

**FIGURE 1:**
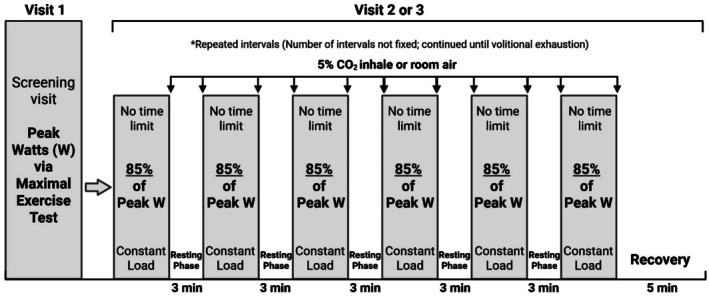
The exercise testing protocol. Participants completed three laboratory visits. (Visit 1) A maximal cardiopulmonary exercise test was performed to determine maximal oxygen consumption (VO_2_max) and peak work rate (Wmax) on a cycle ergometer. Participants who did not achieve VO_2_max exceeding 120% of age‐predicted value were excluded. (Visit 2 and 3) Participants completed two randomized sessions (5% CO_2_ or room air). During each session, participants performed repeated constant‐load cycling at 85% of their Wmax and continued each bout until cadence dropped below 60 rpm or they chose to stop. Participants inhaled 15 breaths of either 5% CO_2_ or room air immediately post‐interval and before initiating new exercise bout during 3‐min resting interval. The number of intervals was not predetermined and was completed until volitional exhaustion.

Subsequently, participants completed an incremental exercise test on an upright cycle ergometer (Excalibur, Lode, Netherlands) to determine VO_2_max and peak work rate (Wmax). The test began with a 2‐min rest at 0 W, followed by a step‐incremental protocol with 3‐min stages. Starting work rate (W) and stage increment were adjusted by sex: males began at 100 W with 40 W increments, and females began at 50 W with 35 W increments. The test continued until volitional exhaustion or failure to maintain cadence above 60 rpm. VO_2_max was defined as the highest 30‐s average VO_2_ achieved. Wmax was calculated as the last completed stage work rate plus a proportional fraction of the final incomplete stage: Wmax = last completed stage W + (time in final stage/180 s) × stage increment W. After completion of the VO_2_max test, participants underwent a familiarization session; a brief period of exercise was completed at 85% of their Wmax after fully recovering from the VO_2_max test. This simulated the bouts that would be completed during the experimental visits (Figure 1).

An intensity of 85% Wmax was selected as it corresponds to the severe‐intensity domain, reliably eliciting rapid oxygen consumption (VO_2_) kinetics, high ventilatory demand, and substantial CO_2_ production (VCO_2_). During such intervals, VCO_2_ rises sharply during exercise and temporarily falls during recovery, creating a window of relative hypocapnia between bouts, a physiological state potentially amenable to correction with brief iCO_2_. A volitional exhaustion‐based interval protocol was employed, as this approach maximizes sensitivity for detecting performance differences between conditions compared to fixed‐duration formats. Moreover, because physiological responses during later intervals are inherently influenced by cumulative fatigue from preceding bouts, examining the first interval in isolation offers a cleaner window into CO_2_'s direct effects on exercise tolerance.

### 2.3 Experimental visits

Of the 11 participants initially enrolled, two were excluded for not achieving a VO_2_max greater than 120% of the age‐predicted value. Consequently, nine participants completed two experimental trials. During each visit, participants performed an interval exercise protocol on an upright bicycle ergometer. The protocol began with 2 min of rest followed by a 2 min warm‐up at 50 W. Participants then exercised at 85% of their peak W until volitional exhaustion or until cadence dropped below 60 rpm. An interval was counted only if the participant sustained the required intensity for at least 2 min; if exhaustion occurred before 2 min had elapsed, the protocol was terminated. Upon completing a valid interval, participants were immediately asked whether they wished to continue. If they chose to proceed, the assigned gas (5% CO_2_ or room air) was administered during the subsequent 3 min active recovery phase at 50 W, and the next interval began thereafter. This sequence of exercise and recovery phase was repeated until participants declined to continue or failed to complete the 2 min minimum.

During the iCO_2_ trial, participants inhaled a 5% CO_2_ gas mixture from a Douglas bag immediately before initiating each exercise phase and again immediately after completing it, prior to the recovery phase. This concentration was selected based on prior study demonstrating measurable physiological responses without adverse effects in healthy individuals (Stepanek et al., 2020; Taylor et al., 2022). Each inhalation bout consisted of 15 breaths without volume control. During the CON trial, room air was provided using an identical method and timing. Cardiorespiratory responses, blood pressure (BP), and perceptual variables were measured throughout each visit as described in Section 2.4.

### 2.4 Outcome variables and instruments

Respiratory gas exchange variables, including VO_2_, VCO_2_, end‐tidal CO_2_ (PETCO_2_), ventilatory equivalent for carbon dioxide (VE/VCO_2_), respiratory rate (RR), respiratory exchange ratio (RER), oxygen pulse (O_2_ pulse), and oxygen uptake efficiency slope (OUES), were measured continuously via a respiratory analysis system (Ultima, MGC Diagnostics, Saint Paul, MN, USA). Heart rate (HR) and peripheral oxygen saturation (SpO_2_) were monitored continuously via a pulse oximeter (Masimo, Irvine, CA, USA). BP was assessed manually by sphygmomanometer at rest, immediate post all intervals (entire exercise phase), and at 1‐min active recovery (50 W) and 2‐min resting recovery (0 W). Rating of perceived exertion (RPE) using the Borg 6–20 scale and dyspnea (DYS) using the modified Borg 0–10 scale were obtained each interval.

### 2.5 Analysis

In the present study, all exercise intervals, except the first interval, were influenced by the preceding interval. For instance, a longer duration in the first interval generally led to a shorter duration in the second interval. Therefore, two separate analyses were conducted: one encompassing all intervals and another focusing solely on the first interval, which was unaffected by prior exertion.

To compare total exercise time, average exercise time per bout, and the total number of bouts, paired sample *t*‐tests were conducted. In addition, due to variations in the number of completed intervals among participants, a repeated‐measures mixed model analysis was performed to examine the distinct effects of CO_2_ versus room air over time during the interval exercise. For the first interval, a repeated‐measures analysis of variance (ANOVA) was conducted to assess changes in BP over time. Subsequently, paired sample *t*‐tests were performed to compare key variables at matched time points near peak and at peak exercise and recovery.

All statistical analyses were conducted using SPSS version 28.0 (IBM Corp., Armonk, NY, USA). Statistical significance was set at *p* < 0.05. Effect sizes were calculated using Hedges' g for outcomes with *p*‐values near the significance threshold.

### 2.6 Patient and public involvement

No patients or members of the public were involved in developing the research question, study design, outcome measures, recruitment, conduct of the study, or dissemination plans. This study involved healthy adult volunteers solely as research participants.

## 3 RESULTS

There was no significant difference in total exercise time between the iCO_2_ and CON trials (24.0 ± 8.1 vs. 23.6 ± 8.6 min). The iCO_2_, however, showed a modest increase in the total number of intervals compared to CON (4.89 ± 1.05 vs. 4.56 ± 1.13, *p* = 0.040, *g* = 0.51), whereas the average time per interval did not differ except for the first interval.

During interval exercise, breathing patterns and respiratory gas‐exchange variables, including peakVO_2_, PETCO_2_, VE/VCO_2_, RR, RER, O_2_pulse and OUES, were not different between trials. Similarly, HR, SpO_2_, RPE, and DYS did not differ across intervals.

During post‐exercise recovery after completion of the entire exercise, systolic blood pressure (SBP) was lower in the iCO_2_ trial than in the CON at 1 min (105.7 ± 17.8 vs. 122 ± 11.7 mmHg, *p* = 0.021) and 2‐min recovery (97.1 ± 11.4 vs. 112 ± 15.8 mmHg, *p* = 0.010). HR was similar between trials at 1‐min recovery (125.6 ± 14.9 vs. 124.34 ± 12.1 bpm) but was nominally higher in the iCO_2_ trial at 2 min recovery (111.1 ± 11.0 vs. 107.2 ± 9.9 bpm, *p* = 0.047, *g* = 0.57).

**FIGURE 2:**
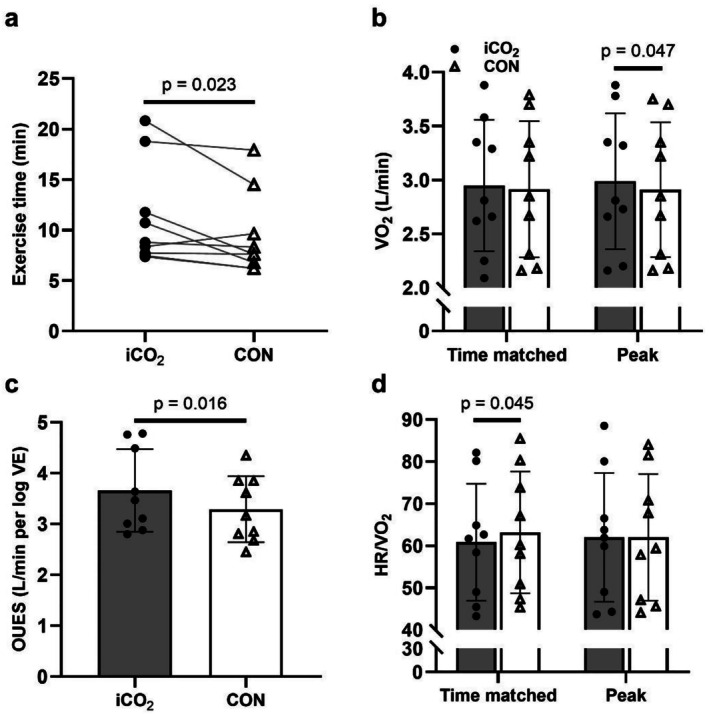
Effects of CO_2_ inhalation (iCO_2_) on first‐interval exercise performance. (a) Total exercise time during the first interval in the iCO_2_ and control (CON) trials. Individual data are shown with connecting lines. (b) Oxygen consumption (VO_2_) at a matched time point and at peak exercise. (c) Oxygen uptake efficiency slope (OUES). (d) Heart rate relative to oxygen consumption (HR/VO_2_) at a matched time point and at peak exercise. Data are presented as mean ± SD.

**FIGURE 3:**
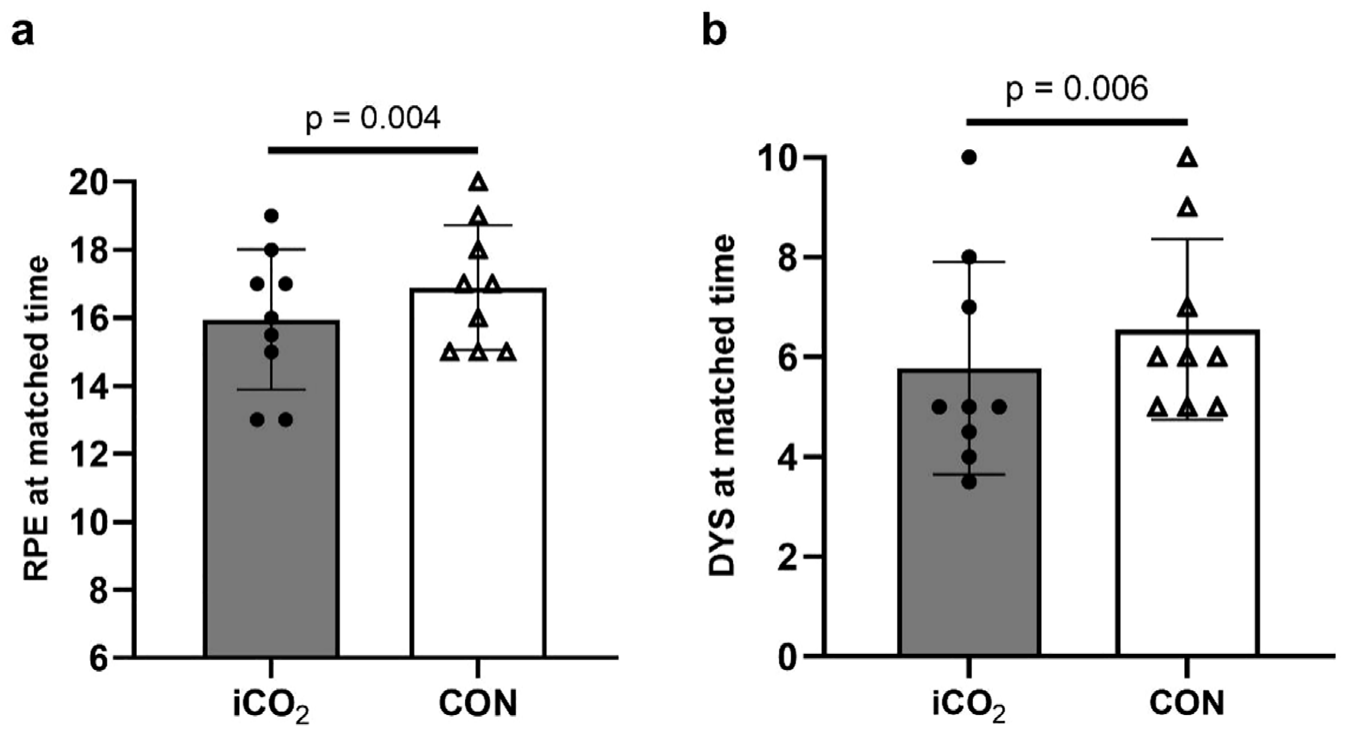
Perceptual responses to CO_2_ inhalation (iCO_2_) during the first interval. (a) Rating of perceived exertion (RPE). (b) Dyspnea score (DYS). Data are presented as mean ± SD.

When only the first interval was analyzed, the iCO_2_ trial demonstrated a longer exercise duration (Figure 2a; *p* = 0.023), a slightly higher peak VO_2_ (Figure 2b; *p* = 0.047, *g* = 0.52), and higher OUES (Figure 2c; *p* = 0.016). However, between trials, there were no significant differences in PETCO_2_, VE/VCO_2_, RR, O_2_ pulse, and RER. Although HR at the matched time point and peak level did not differ, the HR at a given oxygen consumption (HR/VO_2_) at the matched time point during exercise was slightly lower in the iCO_2_ trial (Figure 2d; *p* = 0.045, *g* = −0.56). Furthermore, RPE and DYS were also lower in the iCO_2_ trial at the matched time point relative to CON (Figure 3a,b; *p* = 0.004 and 0.006). During recovery, no significant differences were observed in HR, RPE, and DYS (Table 2).

**TABLE 2:** Differences in key variables at the matched time point near peak level (mchd‐time) and a peak level between CO_2_ inhalation (iCO_2_) and control (CON) conditions during the first interval.

Variables	iCO_2_	CON
*During interval*		
Exercise time (min)	11.3 ± 5.1^a^	9.42 ± 4.1
VCO_2_ (L/min) at beginning exercise	1.42 ± 0.30^a^	1.26 ± 0.17
VCO_2_ (L/min) at peak	3.12 ± 0.78	3.08 ± 0.71
VO_2_ (L/min) at mchd‐time	2.95 ± 0.61	2.91 ± 0.63
VO_2_ (L/min) at peak	2.99 ± 0.63^a^	2.91 ± 0.63
OUES	3.66 ± 0.81^a^	3.29 ± 0.65
RER at mchd‐time	1.04 ± 0.07	1.05 ± 0.04
RER at peak	1.04 ± 0.07	1.04 ± 0.04
VE/VCO_2_ at mchd‐time	35.7 ± 6.3	36.6 ± 5.1
VE/VCO_2_ at peak	37.5 ± 5.7	36.8 ± 5.1
PETCO_2_ (mmHg) at mchd‐time	34.1 ± 6.1	32.9 ± 4.0
PETCO_2_ (mmHg) at peak	32.3 ± 4.4	32.7 ± 4.1
RR (br/min) at mchd‐time	47.6 ± 13.9	48.9 ± 11.6
RR (br/min) at peak	50.9 ± 12.6	49.5 ± 11.2
O_2_ pulse (L/min) at mchd‐time	17.0 ± 4.1	17.0 ± 4.4
O_2_ pulse (L/min) at peak	17.0 ± 4.1	16.7 ± 4.2
HR (bpm) at mchd‐time	174.7 ± 12.2	173.4 ± 15.5
HR (bpm) at peak	176.8 ± 12.2	173.6 ± 15.4
HR/VO_2_ at mchd‐time	60.8 ± 13.9^a^	63.2 ± 14.5
HR/VO_2_ at peak	62.0 ± 15.3	62.5 ± 15.8
SpO_2_ (%) at mchd‐time	97.7 ± 1.7	97.4 ± 1.9
SpO_2_ (%) at peak	97.6 ± 1.5	97.3 ± 1.9
RPE mchd‐time	15.9 ± 2.1^a^	16.9 ± 1.8
RPE at peak	17.7 ± 1.6	17.1 ± 1.8
DYS at mchd‐time	5.8 ± 2.1^a^	6.6 ± 1.8
DYS at peak	7.1 ± 2.1	6.7 ± 1.9
*During recovery*		
HR (bpm)	131.5 ± 13.6	130.4 ± 13.8
SpO_2_ (%)	98.5 ± 1.26	98.2 ± 1.09
RPE	8.89 ± 1.54	9.44 ± 2.19
DYS	1.94 ± 1.07	1.94 ± 0.81

*Note*: Data are presented as mean ± SD. Exercise time measured during the first interval.

Abbreviations: DYS, dyspnea score; HR, heart rate; O_2_ pulse, oxygen pulse; OUES, oxygen uptake efficiency slope; PETCO_2_, end‐tidal carbon dioxide; RER, respiratory exchange ratio; RPE, rating of perceived exertion; RR, respiratory rate; SpO_2_, peripheral oxygen saturation; VCO_2_, carbon dioxide production; VE/VCO_2_, ventilatory equivalent for carbon dioxide; VO_2_, oxygen consumption.

^a^ Significant differences between iCO_2_ and CON conditions (*p* < 0.05).

## 4 DISCUSSION

The present study examined the acute effects of iCO_2_ on performance and recovery during high‐intensity interval exercise. iCO_2_ was associated with a greater number of completed intervals, although total exercise duration did not differ from room air. In the first interval, unaffected by prior fatigue, iCO_2_ resulted in longer exercise duration, lower HR at a given VO_2_ and reduced RPE and DYS values. During post‐exercise recovery, SBP was lower and HR higher under iCO_2_, indicating a distinct cardiovascular response pattern.

iCO_2_ allowed participants to complete a greater number of intervals, yet this advantage did not translate into a longer total exercise duration. This may suggest that iCO_2_ facilitated transient improvements in tolerance or recovery between intervals, rather than producing a sustained enhancement in overall performance. Because physiological responses such as VO_2_, HR and respiratory gas exchange were largely comparable between trials, the performance differences appear subtle and may reflect short‐term modulation rather than a systemic ergogenic effect. Moreover, RPE and DYS were similar across most intervals, indicating that perceptual fatigue and exertional effort were not broadly altered by iCO_2_. One likely explanation is that the duration of each interval was influenced by the preceding bout; participants who performed longer in one interval tended to perform shorter in the next, thus equilibrating total exercise time between trials despite minor intra‐bout advantages.

During the recovery phase after completion of the entire exercise, iCO_2_ elicited a characteristic pattern of reduced SBP and elevated HR. CO_2_ is a well‐known vasodilator that acts partly through nitric oxide‐dependent mechanisms and endothelial relaxation (Chuang et al., 2010; Stepanek et al., 2020). This vasodilation likely lowered vascular resistance and, consequently, SBP. At the same time, hypercapnia is known to activate sympathetic outflow, which may have increased HR via baroreflex compensation (Tymko et al., 2025). The coexistence of vasodilation with sympathetic activation suggests that iCO_2_ modulated both vascular and autonomic control during early recovery. Alternatively, enhanced endothelial nitric oxide synthesis (eNOS) might have temporarily overridden sympathetic vasoconstriction, maintaining vasodilatory tone even after exercise. These findings indicate a complex interplay between CO_2_‐induced vascular effects and autonomic recovery that warrants further investigation.

The first interval offered a clearer view of CO_2_'s potential influence on exercise performance, as it was not confounded by prior fatigue. Here, participants exercised longer and reached slightly higher peak VO_2_ and OUES under iCO_2_. Several physiological mechanisms may contribute to this response. Elevated CO_2_ levels can mitigate exercise‐induced hypocapnia caused by voluntary hyperventilation, thereby reducing pulmonary vasoconstriction and ventilation‐perfusion (V‐Q) mismatch (Crewe et al., 2008; Yilmaz et al., 2025). By shifting the oxyhemoglobin dissociation curve to the right (Bohr effect), CO_2_ may facilitate oxygen unloading and enhance tissue oxygen delivery during intense work (Carr et al., 1993; Woyke et al., 2021). In turn, improved oxygen availability, combined with modestly lower HR at a given VO_2_, could reflect a more efficient cardiovascular response, enabling participants to sustain effort longer. Psychophysiological factors likely contributed as well. Both RPE and DYS were lower at matched near‐peak intensities under iCO_2_, suggesting that reduced perceptual strain may have helped prolong effort (Hashem et al., 2025; Kang et al., 2009). Perceived exertion is known to closely predict endurance capacity, and small reductions can meaningfully delay task termination (Monteiro et al., 2019; Swart et al., 2009). Thus, brief pre‐exercise iCO_2_ may have acted as a form of physiological preconditioning, may adjust metabolic systems, including enhancement of chemoreceptor sensitivity, delaying ventilatory fatigue and increasing cardiovascular reserve (Hashem et al., 2025).

The present findings suggest that brief iCO_2_ may be used as a controlled physiological stimulus to stabilize early ventilatory and perceptual strain during the onset of high‐intensity exercise. By attenuating exercise‐induced hypocapnia and modestly prolonging initial work tolerance, iCO_2_ may help smooth the transition into severe‐intensity workloads, a phase often associated with dyspnea and rapid perceptual escalation. Although the intervention did not enhance total exercise duration, the distinct early‐exercise response profile indicates a potential role for CO_2_ in modulating chemosensory drive and perceptual regulation.

From an applied standpoint, these results highlight the possibility of using inspired‐gas manipulation to influence warm‐up strategies, the initiation phase of interval training, or short‐term recovery between repeated bouts. While direct implementation in athletes or clinical populations is premature, the physiological mechanisms identified here provide a starting point for developing targeted approaches to managing hypocapnia, ventilatory discomfort, or tolerance to intense exercise transitions.

Several limitations should be acknowledged. Mechanistic assessments and autonomic indices were not collected, limiting interpretation of the underlying pathways. The 5% iCO_2_ (15 breaths) was brief, and its dosing and timing may not have elicited cumulative effects through the entire exercise bout. The modest sample size (*n* = 9) restricts statistical power and generalizability; sex‐specific differences in physiological responses were not examined due to the mixed‐sex composition and modest sample size, and potential sex‐related contributions to the observed inter‐individual variability cannot be excluded. The present protocol also differs from conventional high‐intensity interval training formats, which are typically targeted at higher relative intensities (90–100% Wmax) with fixed durations (Buchheit & Laursen, 2013), and whether the current findings extend to such formats remains to be established. Additionally, setting the exercise intensity at 85% Wmax may not have elicited comparable physiological stress across participants, as the relationship between Wmax and critical power varies between individuals (Jamnick et al., 2020), potentially contributing to the observed inter‐individual variability in interval duration. Future studies with larger cohorts and comprehensive physiological measures are warranted to define the dose–response relationship and clarify the integrative cardiovascular and perceptual mechanisms of iCO_2_.

In conclusion, acute 5% iCO_2_ before and after repeated cycling intervals to volitional exhaustion at 85% Wmax modestly influenced performance and cardiovascular recovery. While total exercise time was unchanged, prolonged effort during the first interval and lower perceived exertion at near‐peak intensity suggest that iCO_2_ may facilitate short‐term tolerance and recovery through combined cardiovascular and perceptual mechanisms. These findings should be interpreted within the context of this specific interval format, and further research is needed to examine mechanistic assessments, long‐term effects, optimal dosing and timing, and applications across various interval formats, populations, and exercise modalities.

## AUTHOR CONTRIBUTIONS

**Dongwook Yeo:** Conceptualization; data curation; formal analysis; investigation; methodology; visualization; writing – original Draft. **Jesse C. Schwartz:** Data curation; formal analysis; investigation; resources. **Bruce D. Johnson:** Conceptualization; funding acquisition; supervision; validation. **Chul‐Ho Kim:** Conceptualization; data curation; formal analysis; investigation; supervision; validation; writing – review & editing.

## FUNDING INFORMATION

This study was supported by the Mayo Clinic Cardiovascular Research Center—Preventive Cardiology Program.

## CONFLICT OF INTEREST STATEMENT

The authors declare there are no conflicts of interest related to this work. The funding body had no role in the study design, data collection, analysis, interpretation, or manuscript preparation.

## ETHICS STATEMENT

The study was approved by the institutional Review Board (IRB) of the Mayo Clinic (Protocol No. 23‐012477) and was conducted in accordance with the principles outlined in the Declaration of Helsinki. Written informed consent was obtained from all participants prior to their inclusion in this study. All data were collected and analyzed anonymously. No individual identifiable information is included in this manuscript.

## Data Availability

The data that support the findings of this study are available from the corresponding author upon reasonable request.
